# Muricholic Acids Inhibit *Clostridium difficile* Spore Germination and Growth

**DOI:** 10.1371/journal.pone.0073653

**Published:** 2013-09-09

**Authors:** Michael B. Francis, Charlotte A. Allen, Joseph A. Sorg

**Affiliations:** Department of Biology, Texas A&M University, College Station, Texas, United States of America; Universidad Andres Bello, Chile

## Abstract

Infections caused by *Clostridium difficile* have increased steadily over the past several years. While studies on *C. difficile* virulence and physiology have been hindered, in the past, by lack of genetic approaches and suitable animal models, newly developed technologies and animal models allow these processes to be studied in detail. One such advance is the generation of a mouse-model of *C. difficile* infection. The development of this system is a major step forward in analyzing the genetic requirements for colonization and infection. While important, it is equally as important in understanding what differences exist between mice and humans. One of these differences is the natural bile acid composition. Bile acid-mediated spore germination is an important step in *C. difficile* colonization. Mice produce several different bile acids that are not found in humans. These muricholic acids have the potential to impact *C. difficile* spore germination. Here we find that the three muricholic acids (α-muricholic acid, β-muricholic acid and ω-muricholic acid) inhibit *C. difficile* spore germination and can impact the growth of vegetative cells. These results highlight an important difference between humans and mice and may have an impact on *C. difficile* virulence in the mouse-model of *C. difficile* infection.

## Introduction

*Clostridium difficile* is an anaerobic, spore-forming bacteria that is the leading cause of antibiotic-associated diarrhea. As the costs associated with treatment continue to rise [1], [2], much research has focused on understanding the normal course of infection within humans. One of the challenges in the study of *C. difficile* infections has been developing suitable animal models that adequately reproduce symptoms as presented in humans. Gnotobiotic neonatal piglets, rats, and germ-free mice have all been used to varying degrees of success [3], [4], [5], [6]. The most widely used model has been the Syrian hamster model of *C. difficile* disease [7], [8], [9], [10], [11]. Antibiotic-treated hamsters are very sensitive to *C. difficile* infection with lethal disease presenting approximately 3 days after inoculation by *C. difficile* spores. While the hamster represents an excellent model of acute disease, hamsters typically succumb too quickly to disease to measure factors influencing colonization, representing only the full presentation of disease and not less severe symptoms when exposed to epidemic strains [12]. Such rapid progression of the disease and high mortality can also pose problems when attempting to study relapsing infection.

Several mouse models of infection have been developed [5], [13], [14], [15], [16], [17]. Some of these models use heavy doses of antibiotics (e.g. kanamycin, gentamicin, colistin, metronidazole and vancomycin followed by clindamycin or cefoperazone followed by clindamycin) and then inoculation with *C. difficile* spores or vegetative cells [14], [15]. These antibiotic regimens sensitize mice so that they respond to infection in a dose dependent manner (increasing disease severity with increasing number of dosed cells or spores). Further, antibiotic-treated mice can relapse after a course of antibiotic treatment, to cure the primary infection, and will express some resistance to reinfection when allowed to fully recover from disease [14]. These are important components of an animal model because relapse in humans represents one of the main challenges to current treatment regimens [18], [19].

Because the mouse model is beginning to be a more widely accepted method of testing potential preventative therapies [20] and the genetic requirements for infection [13], [21], [22], it is important to understand what potential variability exists between the mouse model of infection and humans. One potential source of variability is the natural differences between mouse and human microbiota. The use of an antibiotic cocktail before infection is an attempt to impact these other microbes [23], [24]. Another important source of variability is the differences in the natural fecal bile acid composition between mice [25], [26] and humans [27] (and hamsters [28], [29], [30]).

In humans, bile acids are synthesized in the liver as either cholic acid (3α, 7α, 12α-trihydroxy-5β-cholanic acid) or chenodeoxycholic acid [3α, 7α,-dihydroxy-5β-cholanic acid (CDCA)] [27]. These bile acids are then conjugated with either taurine or glycine and, later, further modified by certain members of the colonic microbiota [27]. Previous work has shown that colony formation by *C. difficile* spores on rich medium occurs after exposure to cholic acid derivatives [31], [32]. Subsequent work has shown that all cholic acid derivatives and some amino acids, commonly glycine, can stimulate the initiation of spore germination while CDCA-derivatives are competitive inhibitors of cholic acid-mediated germination [31], [33], [34], [35], [36]. In mice and rats, CDCA is a component of bile, but there are two additional bile acids, α-muricholic acid (AMA) and β-muricholic acid (BMA), that are not present in humans [37]. A third muricholic acid, ω-muricholic acid (OMA), is an epimer of BMA and is produced by the normal microbiota. The effects of these compounds on *C. difficile* spore germination are unknown.

Germination by *C. difficile* spores must be the first step in colonization [20], [38]. The toxins necessary for disease are not found within the spore or deposited on the outer layers during spore formation [39]. To generate active infection in the hamster model of *C. difficile* disease, approximately 100 spores will result in a lethal infection (LD_100_) while in the mouse model, significantly more spores are required to generate lethal disease (∼10^8^) [20]. Interestingly, when vegetative cells are used to inoculate antibiotic-treated mice, fewer cells are needed (∼10^5^), suggesting the efficiency of *in vivo* germination by *C. difficile* spores may be affected differently in the mouse than in the hamster [14], [20], [40].

Here, we investigate how muricholic acids affect *C. difficile* spore germination and growth using two *C. difficile* isolates; UK1– an epidemic ribotype 027 isolate [34], [41] and M68– a ribotype 017 that readily colonizes mice [13], [17]. We find that all three muricholic acids can inhibit *C. difficile* spore germination with apparent affinities similar to what is observed for CDCA and that these compounds are also growth inhibitory.

## Materials and Methods

### *C. difficile* Growth Conditions

*C. difficile* strain UK1 [34], [38], [41], [42] and strain M68 [13], [43] were grown in BHIS medium (Brain Heart Infusion supplemented with 5 g/L yeast extract and 0.1% L-cysteine) at 37°C in an anaerobic environment (85% nitrogen, 10% hydrogen and 5% carbon dioxide).

### *C. difficile* Spore Preparations

Spores of *C. difficile* UK1 and *C. difficile* M68 were prepared as described previously [34], [42], [43]. Briefly, *C. difficile* UK1 or M68 were streaked on BHIS agar medium and incubated for 4 days under anaerobic conditions at 37°C. Plates were then removed from the chamber and cell matter was scraped and diluted into 1 mL of water. Tubes were then left to incubate overnight at 4°C to aid in the release of spores from the mother cell. The next day, cell matter was resuspended and centrifuged at 14,000×g for 1 minute. Tubes were decanted and resuspended in 1 mL of water. After 5 washes, the pellets from several tubes were combined in 2 mL water and layered on top of 8 mL of 50% sucrose. Spores were separated from vegetative cells and cell debris by centrifugation for 20 minutes at 4,000×g. All liquid was then removed from the tube. The pellet, containing the purified spores, was resuspended in 1 mL of water. The purified spores were washed in water as described above. When examined by phase-contrast microscopy, the remaining pellet appeared to be composed >99.9% phase-bright spores.

### Germination of *C. difficile* Spores

Purified spores were heat activated for 30 min at 65°C and placed on ice, as described previously [33], [34], [42], [44], [45]. Heat-activated spores were then diluted into 990 µL BHIS supplemented with 0 mM, 2 mM, 5 mM, 10 mM, 20 mM or 50 mM taurocholate. When testing muricholic acids or CDCA, bile compound was added to tubes before the addition of spores. The initiation of germination was followed by monitoring absorbance at 600 nm. The ratio of the A_600_ at time× (T_x_) to the A_600_ at time zero (T_0_) was plotted against time. Germination rates, and apparent affinities, were determined using the slopes of the linear portions of the germination plots, as described previously [34], [42], [45]. Data are reported as the averages from three independent experiments with one standard deviation from the mean. For clarity, only every fourth data point is plotted. CDCA, AMA, BMA and OMA were dissolved at 100 mM in 100% ethanol. AMA, BMA and OMA were purchased from Steraloids, Inc (Newport, RI).

### Minimum Inhibitory Concentration

*C. difficile,* from an actively growing plate, was grown overnight in 5 mL liquid BHIS under anaerobic conditions. The next day, 25 mL BHIS medium was inoculated with 0.25 mL of the overnight *C. difficile* culture and then incubated until an OD_600_ of 0.45. One hundred twenty five-microliters of this culture then added to 50 mL of ice cold reduced BHIS and kept on ice. Microtiter plates containing BHIS and serially diluted compound were previously prepared and placed in anaerobic chamber to reduce. 10 µL of chilled cells were then added to wells and incubated for 24 hours at 37°C. After 24 hours, plates were removed from the anaerobic chamber and growth measured using a BioRad Xmark plate reader.

#### Statistical significance

Experiments were performed in triplicate and data represent the average of the three independent experiments. Statistical significance between UK1 and M68 was determined using the Student’s T-test.

## Results

### Structures of Muricholic Acids

**Figure 1:**
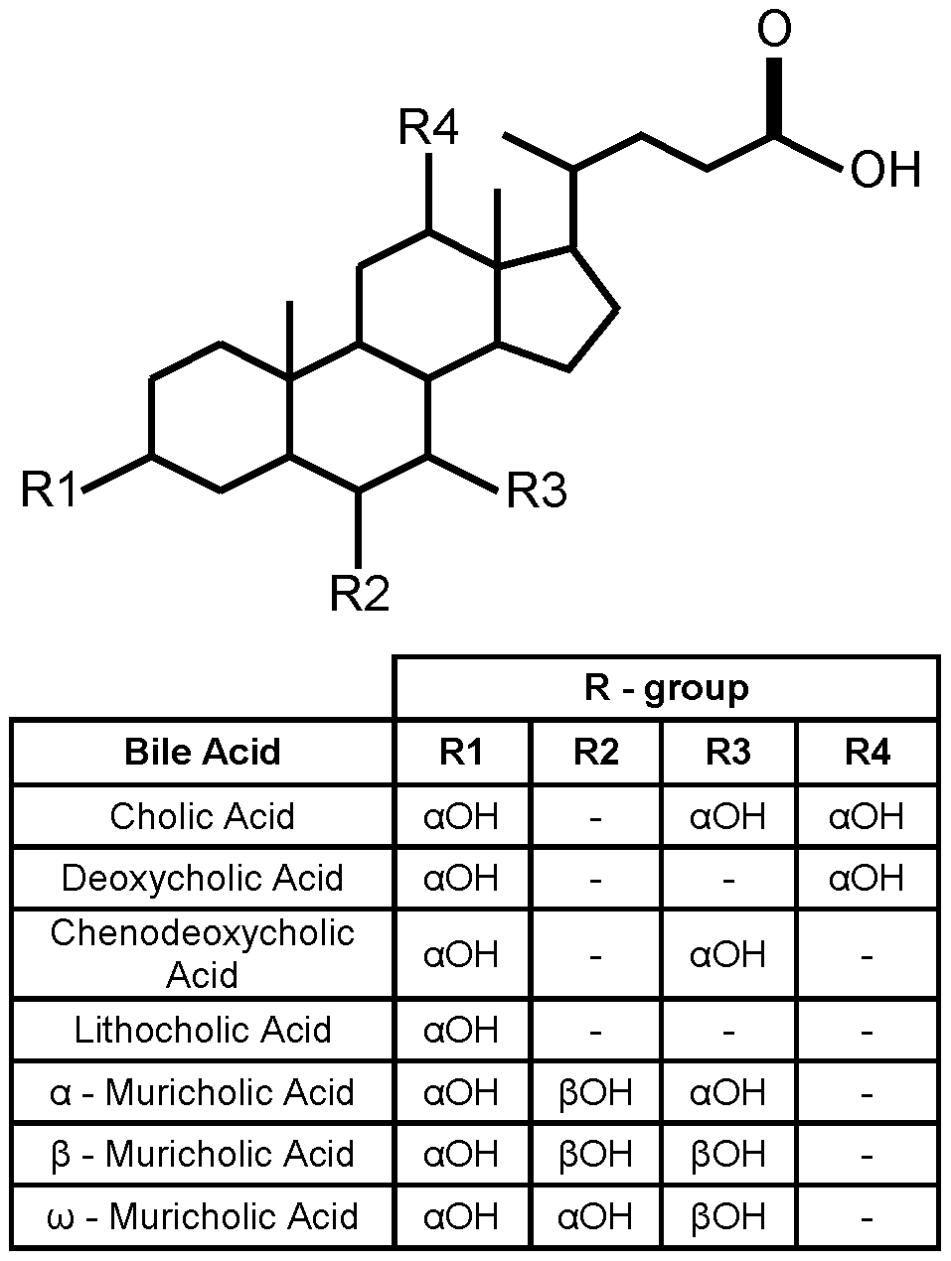
Structures of common muricholic acids. The primary bile acids (cholic acid, chenodeoxycholic acid, α-muricholic acid and β-muricholic acid) are listed. Deoxycholic acid and ω-muricholic acid are secondary bile acids and are products of the normal microbiota.

Mice synthesize three bile acids not found in humans. Two of these compounds are synthesized directly by the mouse; AMA (3α, 6β, 7α-trihydroxy-5β-cholanic acid) and BMA (3α, 6β, 7β-trihydroxy-5β-cholanic acid) (Figure 1) [25], [26]. The third muricholic acid, OMA (3α, 6α, 7β-trihydroxy-5β-cholanic acid) is produced by oxidation of the 6β-hydroxyl of β-muricholic acid followed by reduction of the compound to a 6α-hydroxyl group (Figure 1) by members of the mouse colonic microbiota [46], [47].

AMA and BMA contain a 6β-hydroxyl group while OMA contains a 6α-hydroxyl group (Figure 1). The conformational effect of this 6-hydroxyl group is untested on *C. difficile* spore germination because bile acids normally found in the human gut lack the 6-hydroxyl group. As shown in Figure 1, all three muricholic acids lack a 12α-hydroxyl group, suggesting they might act as inhibitors of *C. difficile* spore germination [34].

### Muricholic Acids Inhibit *C. difficile* Spore Germination

To understand how these compounds affect germination, *C. difficile* spores were assayed for germination in the presence or absence of muricholic acids. As positive and negative controls, respectively, the initiation of spore germination was followed in the presence of taurocholic acid, a known *C. difficile* spore germinant [31], [32] or in the presence of taurocholic acid and CDCA, a known inhibitor of *C. difficile* spore germination [33], [34].

**Figure 2:**
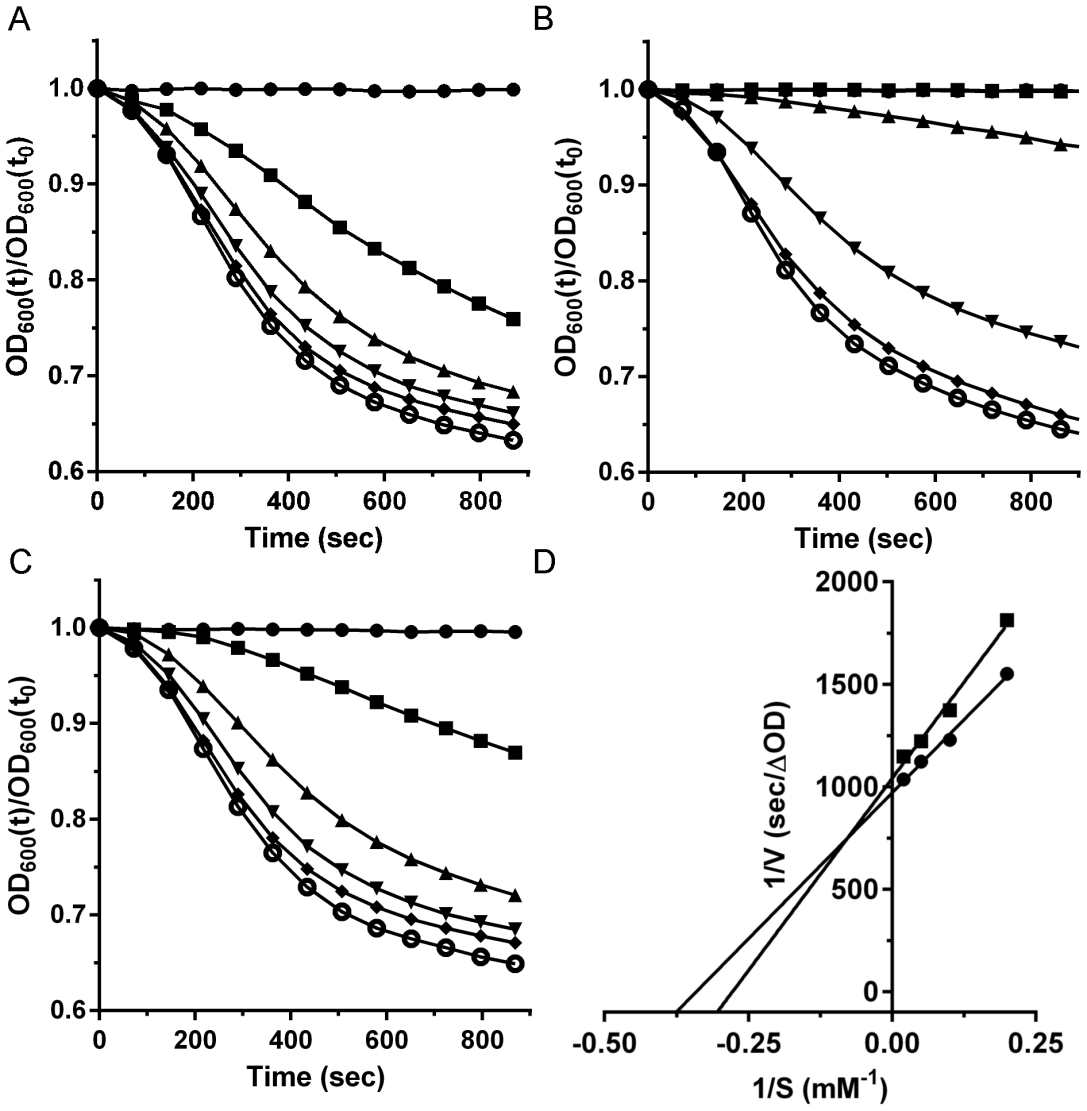
α-muricholic acid inhibits germination by *C. difficile* UK1 spores. (A) Germination of *Clostridium difficile* UK1 spores in complex medium supplemented with taurocholic acid (TA) or (B) medium supplemented with TA and 1 mM CDCA or (C) medium supplemented with TA and 1 mM α-muricholic acid. • 0 mM TA, ▪ 2 mM TA, ▴ 5 mM TA, ▾ 10 mM TA,♦ 20 mM TA or ○ 50 mM TA. (D) The inverse rate (1/*v* [sec/OD_600_]), versus the inverse taurocholate concentration (1/*S* [mM^−1^], was plotted. Apparent K_m_ values for TA alone (•) and in the presence of α-muricholic acid (▪) were determined from the linear best fit.

Purified *C. difficile* UK1 spores were suspended in BHIS medium and different taurocholic acid concentrations (Figure 2A). As described previously, the rate of germination increased with increasing taurocholic acid concentration [34], [42], [45]. The addition of 1 mM CDCA had an inhibitory effect on germination (Figure 2B). The addition of 1 mM AMA resulted in a clear reduction of the ability of *C. difficile* spores to germinate in response to TA (Figure 2C). The effect of this inhibition of germination was quantified by applying Michaelis-Menten kinetics to the germination plots to generate apparent K_m_ values. While not traditional enzyme kinetics, these types of analyses have aided in the identification of the requirements for spore germination and for novel inhibitors of spore germination [34], [36], [42], [44], [45], [48], [49], [50]. Analysis of the Lineweaver-Burk plot of *C. difficile* UK1 spore germination in taurocholic acid alone (Figure 2D) yielded an apparent K_m_ value similar to what has been previously reported (Table 1) [34], [42]. When analyzing germination by *C. difficile* UK1 spores in the presence of different muricholic acids, it was immediately obvious that these compounds were germination-inhibitory. From the germination plots, we determined the rates of germination and used this data to generate apparent inhibitory constants (K_i_) for each inhibitor tested (Table 1). Comparing the muricholic acids to CDCA, BMA and OMA yielded apparent inhibition constants similar to CDCA while AMA proved to be least efficient at inhibiting germination (Table 1). The difference between CDCA and AMA can be observed by comparing the ability of *C. difficile* spores to germinate in BHIS medium supplemented with 2 mM TA. Addition of 1 mM CDCA had a greater effect than did the addition of 1 mM AMA.

**Table 1:** Bile acid effects on *C. difficile* spore germination.

Strain	UK1	M68
	K_m_ (mM)	K_m_ (mM)
Taurocholic Acid	3.2±0.5	3.5±0.5
	K_i_ (mM)	K_i_ (mM)
Chenodeoxycholic Acid	0.22±0.07	0.12±0.02
α-Muricholic Acid	0.62±0.09	0.59±0.05
β-Muricholic Acid	0.27±0.12	0.26±0.02
ω-Muricholic Acid	0.29±0.03	0.20±0.01*

K_i_ = [inhibitor]/[(K_m,TA_ with inhibitor)/((K_m,TA_ without inhibitor) −1)].

* p<0.05.

**Figure 3:**
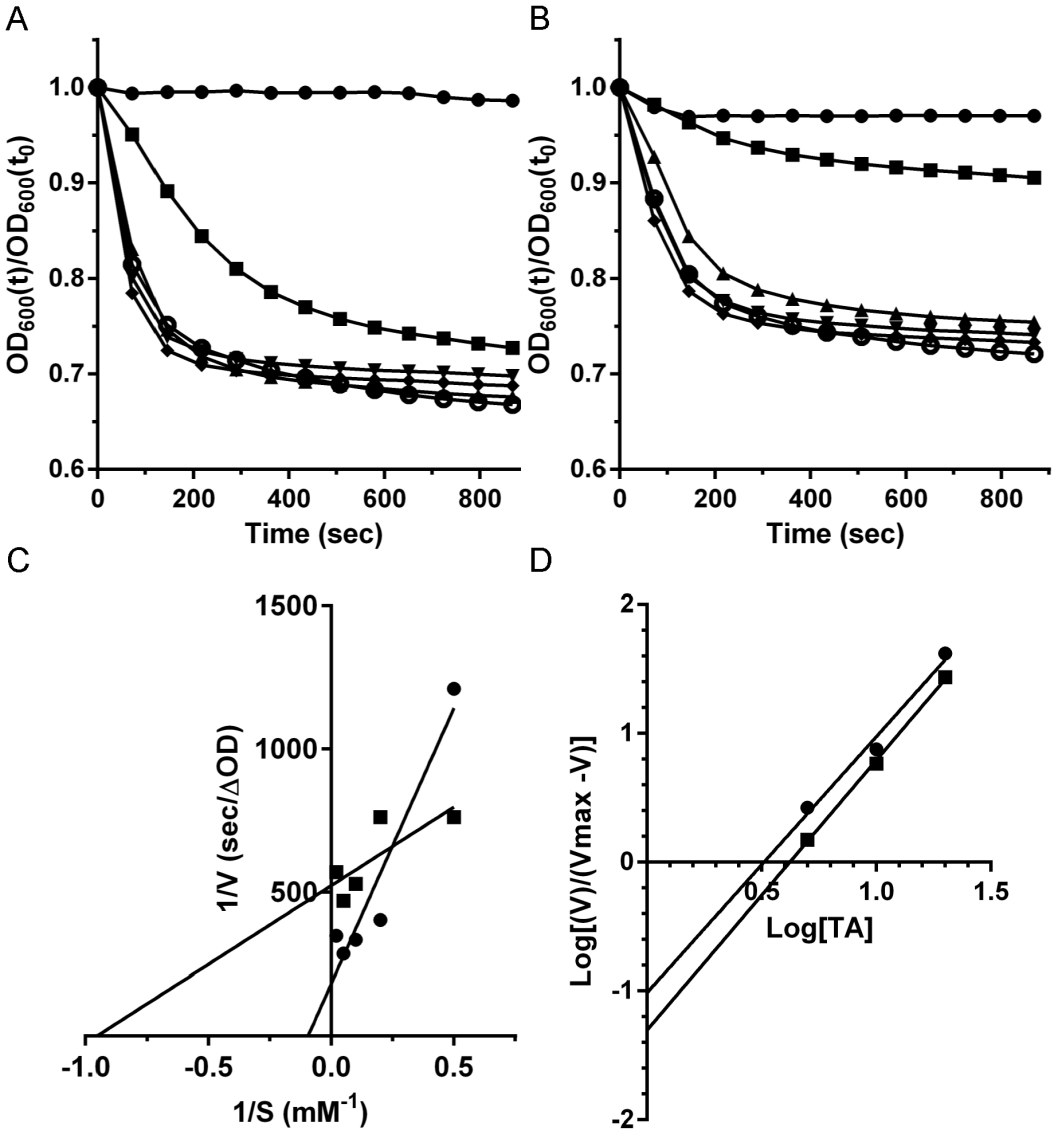
α-muricholic acid inhibits germination by *C. difficile* M68 spores. (A) Germination of *Clostridium difficile* M68 spores in complex medium supplemented with taurocholic acid (TA) or (B) medium supplemented with TA and 1 mM α-muricholic acid. • 0 mM TA, ▪ 2 mM TA, ▴ 5 mM TA, ▾ 10 mM TA,♦ 20 mM TA or ○ 50 mM TA. (C) The inverse rate (1/*v* [sec/OD_600_]), versus the inverse taurocholate concentration (1/*S* [mM^−1^], was plotted. Apparent K_m_ values for TA alone (•) and in the presence of α-muricholic acid (▪) were determined from the linear best fit of the plotted data. (D) Hill Plot was generated to determine the apparent K_m_ values for each condition.

Germination of *C. difficile* M68 in medium with taurocholic acid was similar to *C. difficile* UK1. *C. difficile* M68 rapidly germinated in medium supplemented with taurocholic acid (Figure 3A) and was inhibited when 1 mM AMA was added to the germination solution (Figure 3B). However, by analyzing the kinetics of *C. difficile* M68 spore germination, we observed that the data from this strain produced non-linear Lineweaver-Burk plots (Figure 3C), a phenomenon observed for some other *C. difficile* strains [45]. The Hill plot (Figure 3D) was used to generate the apparent K_m_. This value was then used to determine the apparent K_i_ under each condition tested. When *C. difficile* M68 spores were germinated in the presence of CDCA, germination was strongly inhibited (Table 1). The inhibition of germination by BMA was similar to the inhibition observed for *C. difficile* UK1 and, again, AMA was the least efficient at inhibiting spore germination (Table 1). OMA was a more potent inhibitor of *C. difficile* M68 spore germination than *C. difficile* UK1 spore germination (p-value <0.05).

### Minimum Inhibitory Concentration of Muricholic Bile Acids

Previously, we demonstrated that CDCA and deoxycholic acid inhibited *C. difficile* growth [31]. In antibiotic-treated mice, the levels of deoxycholic acid are likely to be very low because it is a product of the 7α-dehydroxylation of cholic acid by the normal microbiota [27]. However, cholic acid, CDCA, AMA and BMA will be present and could affect *C. difficile* growth. To quantify the effects of these compounds on *C. difficile* growth, we determined the MIC. Serial, 2-fold dilutions of bile acids in growth media were used determine the MIC for each bile acid (Table 2). *C. difficile* strain UK1 did not grow in the presence of CDCA or AMA or BMA, at a concentration of 1 mM or above. OMA was less toxic to the strain; a concentration of 2 mM was necessary to inhibit growth. In stark contrast, the MIC of cholic acid for *C. difficile* UK1 was 10 mM, a concentration not found in the colon. We observed slightly different results when analyzing the MIC of these bile acids for *C. difficile* M68 growth. This strain was more resistant to the toxic effects of AMA and BMA while equally as sensitive to CDCA, cholic acid and deoxycholic acid (Table 2). BMA is more prevalent in the gut of rats and mice than is CDCA [25], [51], suggesting that a strain which is more resistant to the toxic effects of BMA (e.g. *C. difficile* M68) might be able to better colonize mice.

**Table:** Table 2. Minimum inhibitory concentration of bile acids for *C. difficile* strains.

Strain	UK1 (mM)	M68 (mM)
Cholic Acid	10.0±0.0	10.0±0.0
Deoxycholic Acid	1.0±0.0	1.0±0.0
Chenodeoxycholic Acid	1.0±0.0	1.0±0.0
α-Muricholic Acid*	1.0±0.0	2.0±0.0
β-Muricholic Acid*	1.0±0.0	2.0±0.0
ω-Muricholic Acid	2.0±0.0	2.0±0.0

The MIC did not vary between experiments.

* p<0.01.

## Discussion

In the laboratory setting, certain combinations of bile acids and amino acids are the most effective conditions for measuring *C. difficile* spore germination [31], [32], [35]. While cholic acid derivatives can stimulate *C. difficile* spore germination [31], CDCA-derivatives inhibit cholic acid-mediate germination by *C. difficile* spores [33], [34]. Compared to humans, mice produce a low level of CDCA but produce other bile acids (AMA and BMA), in greater abundance. These muricholic acids may have an impact on how *C. difficile* spores germinate *in vivo*. Here, we found that AMA, BMA and OMA (a microbial product) inhibit taurocholic acid-mediated spore germination with BMA and OMA being the most potent germination-inhibiting muricholic acids (Table 1). These results are consistent with our previous work that has shown the 12-hydroxyl group to be an important determinant of whether a compound functions as a germinant or inhibitor of germination [31], [33], [34]. One difference observed between the germination of *C. difficile* UK1 spores and *C. difficile* M68 spores was the non-linear double-reciprocal plot for germination by *C. difficile* M68 spores. As seen in other strains, *C. difficile* M68 may bind taurocholic acid cooperatively [45]. With the recent identification of the molecular target of bile acids on the *C. difficile* spore, this hypothesis could be tested outright [38].

Total bile acid levels in the distal small intestine have been estimated to be between 1 mM to 2 mM in concentration [52]. This is in the range of the concentrations which inhibit *C. difficile* growth for the individual bile acids tested (Table 2); variations in pH may affect the toxicity of each bile acid [53]. Comparing these concentrations to the apparent K_i_ values determined for AMA and BMA, they are approximately 3× to 8× greater, respectively (Table 1). That is, in an antibiotic-treated mouse, the levels of AMA and BMA might prevent efficient *C. difficile* spore germination, possibly explaining why such greater numbers of spores, compared to vegetative cells, are required to colonize a mouse [20]. It is also important to note that most mice used as a model for *C. difficile* infection would likely contain reduced levels of OMA because its formation requires the presence of mouse gut microbes [46], [47] which are likely ‘collateral damage’ during a routine course of broad-spectrum antibiotics.

Antibiotics can affect host functions. That is, treating mice with antibiotics could lead to alterations in the bile acid spectrum and increase or decrease the availability of activators or inhibitors of *C. difficile* spore germination. Treatment of mice with antibiotics has been shown to increase hepatic bile acid synthesis [54]. Specifically, the authors identified that small intestine, lumenal concentrations of taurocholic acid, tauro-β-muricholic acid and taurochenodeoxycholic acid were more abundant in antibiotic-treated C57/BL6 mice than in vehicle-only controls [54]; the authors did not measure the levels of AMA. Thus, upon antibiotic exposure, an increase in the abundance of germination-inhibiting bile acids could contribute to an environment which is more resistant to *C. difficile* spore germination.

Some *C. difficile* strains have been shown to stably colonize mice and enter a ‘contagious’ state, where disease is limited but spore shedding is maintained, while other strains are cleared by the host [13], [17]. The mechanisms by which some *C. difficile* strains are able to stably colonize a host while others do not, is unclear. While the answer is likely to be multifactorial, an increased resistance to bile acids could contribute to a strain’s ability to persist within a host. *C. difficile* M68 is a strain that can enter a supershedder state after the cessation of antibiotic treatment [13]. We find *C. difficile* M68 to be more resistant to bile acid toxicity than is *C. difficile* UK1 and this increased resistance may aid *C. difficile* M68 in maintaining active colonization.

Muricholic acids might provide a level of protection to mice from *C. difficile* infection that is not seen in other models of *C. difficile* disease. While our results suggest that particular bile acids may inhibit *C. difficile* spore germination or vegetative growth *in vitro*, it is unclear if AMA or BMA could substitute for each other in preventing *in vivo* spore germination. Clearly, BMA is a more potent inhibitor of *in vitro* spore germination than is AMA. But, given the vast repertoire of mouse lines and genetic approaches, testing the ability of *C. difficile* to colonize mice that have had introduced mutations into specific steps in the bile acid/muricholic acid synthesis pathway would allow the determination of which bile acids are relevant for stimulating or inhibiting *in vivo* spore germination and vegetative growth.

## References

[1] Kyne L, Hamel MB, Polavaram R, Kelly CP (2002) Health care costs and mortality associated with nosocomial diarrhea due to *Clostridium difficile* . Clin Infect Dis 34: 346–353. 11774082 10.1086/338260

[2] O’Brien JA, Lahue BJ, Caro JJ, Davidson DM (2007) The emerging infectious challenge of *Clostridium difficile*-associated disease in Massachusetts hospitals: clinical and economic consequences. Infect Control Hosp Epidemiol 28: 1219–1227. 17926270 10.1086/522676

[3] Czuprynski CJ, Johnson WJ, Balish E, Wilkins T (1983) Pseudomembranous colitis in *Clostridium difficile*-monoassociated rats. Infect Immun 39: 1368–1376. 6840842 10.1128/iai.39.3.1368-1376.1983 PMC348107

[4] Reeves AE, Koenigsknecht MJ, Bergin IL, Young VB (2012) Suppression of *Clostridium difficile* in the gastrointestinal tracts of germfree mice inoculated with a murine isolate from the family Lachnospiraceae. Infect Immun 80: 3786–3794. 22890996 10.1128/IAI.00647-12 PMC3486043

[5] Pawlowski SW, Calabrese G, Kolling GL, Platts-Mills J, Freire R, et al (2010) Murine model of *Clostridium difficile* infection with aged gnotobiotic C57BL/6 mice and a BI/NAP1 strain. J Infect Dis 202: 1708–1712. 20977342 10.1086/657086 PMC3057484

[6] Steele J, Feng H, Parry N, Tzipori S (2010) Piglet models of acute or chronic *Clostridium difficile* illness. J Infect Dis 201: 428–434. 20039803 10.1086/649799 PMC2804769

[7] Bartlett JG, Chang BJ, Moon N, Onderdonk AB (1978) Antibiotic-induced lethal entercolitis in hamsters: studies with eleven agents and evidence to support the pathogenic role of toxin-producing Clostridia. Am J Vet Res 39: 1525–1530. 697162

[8] Fekety FR, Silva J, Toshniwal R, Allo M, Armstrong J, et al (1979) Antibiotic-associated colitis: effects of antibiotics on *Clostridium difficile* and the disease in hamsters. Rev Infect Dis 1: 386–397. 549190 10.1093/clinids/1.2.386

[9] Sambol SP, Tang JK, Merrigan MM, Johnson S, Gerding DN (2001) Infection of hamsters with epidemiologically important strains of *Clostridium difficile.* . J Infect Dis 183: 1760–1766. 11372028 10.1086/320736

[10] Kuehne SA, Cartman ST, Heap JT, Kelly ML, Cockayne A, et al (2010) The role of toxin A and toxin B in *Clostridium difficile* infection. Nature 467: 711–713. 20844489 10.1038/nature09397

[11] Lyras D, O’Connor JR, Howarth PM, Sambol SP, Carter GP, et al. (2009) Toxin B is essential for virulence of *Clostridium difficile*. Nature doi:10.1038/nature07822. 10.1038/nature07822 PMC2679968 19252482

[12] Borriello SP, Ketley JM, Mitchell TJ, Barclay FE, Welch AR, et al (1987) *Clostridium difficile*–a spectrum of virulence and analysis of putative virulence determinants in the hamster model of antibiotic-associated colitis. J Med Microbiol 24: 53–64. 3612744 10.1099/00222615-24-1-53

[13] Lawley TD, Clare S, Walker AW, Goulding D, Stabler RA, et al (2009) Antibiotic treatment of *Clostridium difficile* carrier mice triggers a supershedder state, spore-mediated transmission, and severe disease in immunocompromised hosts. Infect Immun 77: 3661–3669. 19564382 10.1128/IAI.00558-09 PMC2737984

[14] Chen X, Katchar K, Goldsmith JD, Nanthakumar N, Cheknis A, et al (2008) A mouse model of *Clostridium difficile*-associated disease. Gastroenterology 135: 1984–1992. 18848941 10.1053/j.gastro.2008.09.002

[15] Reeves AE, Theriot CM, Bergin IL, Huffnagle GB, Schloss PD, et al (2011) The interplay between microbiome dynamics and pathogen dynamics in a murine model of *Clostridium difficile* Infection. Gut Microbes 2: 145–158. 21804357 10.4161/gmic.2.3.16333 PMC3225775

[16] Buffie CG, Jarchum I, Equinda M, Lipuma L, Gobourne A, et al (2012) Profound alterations of intestinal microbiota following a single dose of clindamycin results in sustained susceptibility to *Clostridium difficile*-induced colitis. Infect Immun 80: 62–73. 22006564 10.1128/IAI.05496-11 PMC3255689

[17] Lawley TD, Clare S, Walker AW, Stares MD, Connor TR, et al (2012) Targeted restoration of the intestinal microbiota with a simple, defined bacteriotherapy resolves relapsing *Clostridium difficile* disease in mice. PLoS Pathog 8: e1002995. 23133377 10.1371/journal.ppat.1002995 PMC3486913

[18] Marsh JW, Arora R, Schlackman JL, Shutt KA, Curry SR, et al. (2012) Recurrent *Clostridium difficile* Disease: Association of Relapse with BI/NAP1/027. J Clin Microbiol. 10.1128/JCM.02291-12 PMC3502988 23052318

[19] van Nood E, Vrieze A, Nieuwdorp M, Fuentes S, Zoetendal EG, et al (2013) Duodenal infusion of donor feces for recurrent *Clostridium difficile* . N Engl J Med 368: 407–415. 23323867 10.1056/NEJMoa1205037

[20] Howerton A, Patra M, Abel-Santos E (2013) A new strategy for the prevention of *Clostridium difficile* infections. J Infect Dis. 10.1093/infdis/jit068 23420906

[21] Jarchum I, Liu M, Lipuma L, Pamer EG (2011) Toll-like receptor 5 stimulation protects mice from acute *Clostridium difficile* colitis. Infect Immun 79: 1498–1503. 21245274 10.1128/IAI.01196-10 PMC3067529

[22] Jarchum I, Liu M, Shi C, Equinda M, Pamer EG (2012) Critical role for MyD88-mediated neutrophil recruitment during *Clostridium difficile* colitis. Infect Immun 80: 2989–2996. 22689818 10.1128/IAI.00448-12 PMC3418725

[23] Antonopoulos DA, Huse SM, Morrison HG, Schmidt TM, Sogin ML, et al (2009) Reproducible community dynamics of the gastrointestinal microbiota following antibiotic perturbation. Infect Immun 77: 2367–2375. 19307217 10.1128/IAI.01520-08 PMC2687343

[24] Britton RA, Young VB (2012) Interaction between the intestinal microbiota and host in *Clostridium difficile* colonization resistance. Trends Microbiol 20: 313–319. 22595318 10.1016/j.tim.2012.04.001 PMC3408078

[25] Alnouti Y, Csanaky IL, Klaassen CD (2008) Quantitative-profiling of bile acids and their conjugates in mouse liver, bile, plasma, and urine using LC-MS/MS. J Chromatogr B Analyt Technol Biomed Life Sci 873: 209–217. 10.1016/j.jchromb.2008.08.018 PMC2582521 18801708

[26] Eyssen H, Smets L, Parmentier G, Janssen G (1977) Sex-linked differences in bile acid metabolism of germfree rats. Life Sci 21: 707–712. 904444 10.1016/0024-3205(77)90079-0

[27] Ridlon JM, Kang D, Hylemon PB (2006) Bile salt biotransformations by human intestinal bacteria. J Lipid Res 47: 241–259. 16299351 10.1194/jlr.R500013-JLR200

[28] Hong YJ, Turowski M, Lin JT, Yokoyama WH (2007) Simultaneous characterization of bile acid, sterols, and determination of acylglycerides in feces from soluble cellulose-fed hamsters using HPLC with evaporative light-scattering detection and APCI-MS. J Agric Food Chem 55: 9750–9757. 17979236 10.1021/jf071798+

[29] Benson GM, Haskins NJ, Eckers C, Moore PJ, Reid DG, et al (1993) Polydeoxycholate in human and hamster feces: a major product of cholate metabolism. J Lipid Res 34: 2121–2134. 8301231

[30] Une M, Yamanaga K, Mosbach EH, Tsujimura K, Hoshita T (1990) Metabolism of 7 beta-alkyl chenodeoxycholic acid analogs and their effect on cholesterol metabolism in hamsters. J Lipid Res 31: 1015–1021. 2373951

[31] Sorg JA, Sonenshein AL (2008) Bile salts and glycine as cogerminants for *Clostridium difficile* spores. J Bacteriol 190: 2505–2512. 18245298 10.1128/JB.01765-07 PMC2293200

[32] Wilson KH, Kennedy MJ, Fekety FR (1982) Use of sodium taurocholate to enhance spore recovery on a medium selective for *Clostridium difficile* . J Clin Microbiol 15: 443–446. 7076817 10.1128/jcm.15.3.443-446.1982 PMC272115

[33] Sorg JA, Sonenshein AL (2009) Chenodeoxycholate is an inhibitor of *Clostridium difficile* spore germination. J Bacteriol 191: 1115–1117. 19060152 10.1128/JB.01260-08 PMC2632082

[34] Sorg JA, Sonenshein AL (2010) Inhibiting the initiation of *Clostridium difficile* spore germination using analogs of chenodeoxycholic acid, a bile acid. J Bacteriol 192: 4983–4990. 20675492 10.1128/JB.00610-10 PMC2944524

[35] Wheeldon LJ, Worthington T, Hilton AC, Elliot TS, Lambert PA (2008) Physical and chemical factors influencing the germination of *Clostridium difficile* spores. Journal of Applied Microbiology 105: 2223–2230. 19120667 10.1111/j.1365-2672.2008.03965.x

[36] Howerton A, Ramirez N, Abel-Santos E (2011) Mapping interactions between germinants and *Clostridium difficile* spores. J Bacteriol 193: 274–282. 20971909 10.1128/JB.00980-10 PMC3019946

[37] Hofmann AF (1999) The continuing importance of bile acids in liver and intestinal disease. Arch Intern Med 159: 2647–2658. 10597755 10.1001/archinte.159.22.2647

[38] Francis MB, Allen CA, Shrestha R, Sorg JA (2013) Bile Acid Recognition by the *Clostridium difficile* Germinant Receptor, CspC, Is Important for Establishing Infection. PLoS Pathog 9: e1003356. 23675301 10.1371/journal.ppat.1003356 PMC3649964

[39] Lawley TD, Croucher NJ, Yu L, Clare S, Sebaihia M, et al (2009) Proteomic and genomic characterization of highly infectious *Clostridium difficile* 630 spores. J Bacteriol 191: 5377–5386. 19542279 10.1128/JB.00597-09 PMC2725610

[40] Larson HE, Borriello SP (1990) Quantitative study of antibiotic-induced susceptibility to *Clostridium difficile* enterocecitis in hamsters. Antimicrob Agents Chemother 34: 1348–1353. 2386366 10.1128/aac.34.7.1348 PMC175979

[41] Killgore G, Thompson A, Johnson S, Brazier J, Kuijper E, et al (2008) Comparison of seven techniques for typing international epidemic strains of *Clostridium difficile*: restriction endonuclease analysis, pulsed-field gel electrophoresis, PCR-ribotyping, multilocus sequence typing, multilocus variable-number tandem-repeat analysis, amplified fragment length polymorphism, and surface layer protein A gene sequence typing. J Clin Microbiol 46: 431–437. 18039796 10.1128/JCM.01484-07 PMC2238077

[42] Allen CA, Babakhani F, Sears P, Nguyen L, Sorg JA (2013) Both Fidaxomicin and Vancomycin Inhibit Outgrowth of *Clostridium difficile* Spores. Antimicrob Agents Chemother 57: 664–667. 23147724 10.1128/AAC.01611-12 PMC3535933

[43] Drudy D, Harnedy N, Fanning S, O’Mahony R, Kyne L (2007) Isolation and characterisation of toxin A-negative, toxin B-positive *Clostridium difficile* in Dublin, Ireland. Clin Microbiol Infect 13: 298–304. 17391385 10.1111/j.1469-0691.2006.01634.x

[44] Liggins M, Ramirez N, Magnuson N, Abel-Santos E (2011) Progesterone analogs influence germination of *Clostridium sordellii* and *Clostridium difficile* spores in vitro. J Bacteriol 193: 2776–2783. 21478359 10.1128/JB.00058-11 PMC3133115

[45] Ramirez N, Liggins M, Abel-Santos E (2010) Kinetic evidence for the presence of putative germination receptors in *Clostridium difficile* spores. J Bacteriol 192: 4215–4222. 20562307 10.1128/JB.00488-10 PMC2916422

[46] Eyssen H, De Pauw G, Stragier J, Verhulst A (1983) Cooperative formation of omega-muricholic acid by intestinal microorganisms. Appl Environ Microbiol 45: 141–147. 6824314 10.1128/aem.45.1.141-147.1983 PMC242244

[47] Eyssen HJ, De Pauw G, Van Eldere J (1999) Formation of hyodeoxycholic acid from muricholic acid and hyocholic acid by an unidentified gram-positive rod termed HDCA-1 isolated from rat intestinal microflora. Appl Environ Microbiol 65: 3158–3163. 10388717 10.1128/aem.65.7.3158-3163.1999 PMC91470

[48] Akoachere M, Squires RC, Nour AM, Angelov L, Brojatsch J, et al (2007) Indentification of an *in vivo* inhibitor of *Bacillus anthracis* spore germination. J Biol Chem 282: 12112–12118. 17296608 10.1074/jbc.M611432200

[49] Ramirez N, Abel-Santos E (2010) Requirements for germination of *Clostridium sordellii* spores in vitro. J Bacteriol 192: 418–425. 19915025 10.1128/JB.01226-09 PMC2805323

[50] Dodatko T, Akoachere M, Jimenez N, Alvarez Z, Abel-Santos E (2010) Dissecting interactions between nucleosides and germination receptors in *Bacillus cereus* 569 spores. Microbiology 156: 1244–1255. 20035009 10.1099/mic.0.030270-0 PMC2889443

[51] Alnouti Y, Csanaky IL, Klaassen CD (2008) Quantitative-profiling of bile acids and their conjugates in mouse liver, bile, plasma, and urine using LC–MS/MS. Journal of Chromatography B 873: 209–217. 10.1016/j.jchromb.2008.08.018 PMC2582521 18801708

[52] Northfield T, McColl I (1973) Postprandial concentrations of free and conjugated bile acids down the length of the normal human small intestine. Gut 14: 513–518. 4729918 10.1136/gut.14.7.513 PMC1412809

[53] Hamilton JP, Xie G, Raufman J-P, Hogan S, Griffin TL, et al (2007) Human cecal bile acids: concentration and spectrum. Am J Physiol Gastrointest Liver Physiol 293: G256–263. 17412828 10.1152/ajpgi.00027.2007

[54] Miyata M, Takamatsu Y, Kuribayashi H, Yamazoe Y (2009) Administration of ampicillin elevates hepatic primary bile acid synthesis through suppression of ileal fibroblast growth factor 15 expression. J Pharmacol Exp Ther 331: 1079–1085. 19767447 10.1124/jpet.109.160093

